# Preparing Newly Qualified Doctors for Digital Practice: Addressing the Digital Training Disparity in Medical Education

**DOI:** 10.7759/cureus.111480

**Published:** 2026-06-25

**Authors:** Nauman Khan, Ethel Gomez Canales, Komal Khan, Sarvjit S Sahedra

**Affiliations:** 1 Geriatrics and Acute Medicine, Northampton General Hospital NHS Trust, Northampton, GBR; 2 Geriatric Medicine, Great Western Hospitals NHS Foundation Trust, Swindon, GBR; 3 Radiation Oncology, The Christie NHS Foundation Trust, Manchester, GBR; 4 General Internal Medicine, University Hospitals of Leicester NHS Trust, Leicester, GBR

**Keywords:** digital health education, electronic health records, hospital information system (his), undergraduate medical curriculum, undergraduate medical student

## Abstract

Hospital information systems (HIS) are now integral to the modern healthcare domain. These systems not only record the data in the form of clinical notes but also make the whole process of patient care easy, utilising electronic prescriptions, accessing laboratory results, previous clinical records, and much more. The list of the significance of these digital systems is non-exhaustive. Despite their widespread use in clinical practice, formal education on HIS remains limited within undergraduate medical training. As a result, newly qualified doctors often enter practice with little preparation for the digital environments in which patient care is delivered. This editorial not only explores the reasons for this gap, including traditional curriculum structures, limited faculty expertise, increasing curricular demands, and the assumption that digital competencies can be acquired during clinical practice, but also intends to highlight the need for curriculum revision. It also considers the implications for physician readiness, patient safety, and quality of care, and argues that competency in HIS should be regarded as a fundamental outcome of undergraduate medical education. While core competencies are transferable across healthcare settings, the diversity of HIS adopted by different hospitals and healthcare regions presents an additional challenge, requiring clinicians to adapt to local software platforms and workflows. This also highlights the use of various software due to the market’s influence.

## Editorial

The delivery of healthcare has undergone a significant transformation over recent decades, with digital technologies now embedded in almost every aspect of clinical practice. In many healthcare settings, patient care is closely intertwined with hospital information systems (HIS), which support activities such as reviewing laboratory results, ensuring data safety, prescribing medications, documenting consultations, and requesting investigations. Despite their central role in daily clinical work, there is no formal undergraduate curriculum comparable to traditional subjects such as anatomy, physiology, pathology, pharmacology, medicine, surgery, communication, and clinical skills. This may leave new graduates less prepared to navigate digitally enabled clinical environments safely and efficiently during their transition to professional practice. Although HIS varies across institutions and healthcare systems, these competencies could potentially be developed through placement-based learning integrated within clinical environments. However, further work is required to determine the most effective implementation strategies, learning outcomes, and assessment methods. This may leave new graduates less prepared to navigate electronically enabling clinical environments safely and efficiently during their transition to professional practice. While the primary focus of this editorial is undergraduate medical education, it is also important to recognise that experienced physicians who trained before the widespread adoption of HIS may benefit from structured training and adequate time to become familiar with these technologies.

This disparity raises important questions about how effectively medical education prepares students for contemporary practice. Although this editorial focuses on undergraduate medical education, the need for HIS training extends beyond medical students and newly qualified doctors. Older physicians who trained before the widespread adoption of these systems may also benefit from structured and potentially mandatory training to support the effective use of HIS and ongoing professional practice. Although undergraduate curricula devote substantial time to traditional medical subjects, many graduates begin their first clinical posts with limited experience of electronic health records (EHRs). Consequently, newly qualified doctors must learn to navigate these systems while simultaneously managing the transition from medical student to practising doctor. Although induction programmes may provide a brief introduction to these systems, the limited duration of such training cannot be compared with the extensive time devoted to undergraduate medical education. This gap has already been recognised in the literature with some suggestions. While the focus of this editorial is the preparedness of newly qualified doctors entering clinical practice, digital health training is also relevant to more experienced physicians who may have had limited exposure to HIS during their formal training. An International Consensus Statement based on a Delphi study not only highlighted the importance of worldwide digital education for medical students and junior doctors but also emphasised the imperative for more training in this domain [1].

The persistence of this gap is notable given the extent to which digital technologies now shape healthcare delivery. Newly qualified doctors may spend a considerable proportion of their working day interacting with electronic systems, making digital competence increasingly relevant to routine clinical practice. Nevertheless, competence in this system is frequently viewed as a workplace skill acquired through local induction rather than a core professional competency requiring structured educational preparation. Although medical students spend considerable time in clinical environments and at the bedside, opportunities to engage with the digital infrastructure underpinning patient care are often overlooked. Integrating a structured system within clinical placements may provide students with the opportunity to develop familiarity with HIS in a real-world clinical context, which also includes various market searches regarding software, without requiring substantial additional curriculum time. Students are often trained extensively for bedside practice but receive limited exposure to the digital infrastructure that increasingly supports patient care, and reliance on workplace exposure alone can result in inconsistent levels of competence. Similar challenges may also be encountered across the wider medical workforce, highlighting the importance of digital health education and training throughout a clinician’s career. The consequences of inadequate preparation regarding HIS are increasingly apparent. Fresh graduates commonly report difficulties adapting to unfamiliar electronic systems during their early months of practice. This issue was further highlighted in a mixed-methods survey conducted among medical students from 39 European countries. The study found that 53.2% of participants rated their eHealth Skills as poor or very poor and considered the lack of education in this domain as a main reason. In comparison, 84.9% agreed or strongly agreed that more education should be incorporated into the medical curriculum [2].

Inadequate preparation for digital clinical environments may contribute to difficulties in prescribing, documentation, order entry, and the appropriate management of decision-support alerts, although these outcomes are also influenced by system design, workflow, and organisational factors. Such challenges may contribute to inefficiencies, treatment delays, and potential risks to patient care. Furthermore, growing concerns surrounding cybersecurity and data protection require clinicians to possess these competencies that extend beyond basic system navigation. Collectively, these factors make newly qualified medical graduates particularly vulnerable in an increasingly advanced digital healthcare environment. The underlying reason remains consistent: insufficient exposure towards training in HIS at the undergraduate level. This was further reinforced by a systematic review published in January 2020, examining the training of medical students and residents in the use of EHRs. The review not only highlighted the necessity of formal EHR training but also identified a limited number of studies addressing this important area of medical education [3].

Faculty expertise presents an additional challenge, while curriculum overcrowding is frequently cited as a barrier to introducing additional curriculum. Medical schools face increasing pressure to incorporate emerging subjects such as artificial intelligence, planetary health, and health equity. However, HIS differs from many other curricular topics because its relevance extends across all specialities and practice settings. Regardless of career choice, every medical graduate will use HIS daily, and this may become more extensive, working with artificial intelligence and similar software to create an effective clinical environment. This universal applicability further strengthens the argument for incorporating these competencies as a core component of undergraduate medical education. The importance of such training was also recognised earlier in 2014 when EHR training in undergraduate medical education was described as an educational innovation. This highlighted the growing need to prepare future doctors for the increasing integration of HIS within healthcare systems [4]. This need was further demonstrated by a cross-sectional survey conducted in China in 2023, which highlighted the need for digital health education among the next generation of healthcare professionals. The study revealed that the digital health educational needs of Chinese medical students were not being sufficiently met. Notably, the survey included 2,122 valid responses from students across 467 medical schools, providing substantial evidence of the scale and significance of this unmet need. This study further argued for a national initiative in this area [5].

In conclusion, HIS has become an essential component of modern healthcare delivery, yet formal training in its use remains insufficient within many undergraduate medical curricula. As healthcare continues to evolve in an increasingly digital environment, integrating structured HIS into existing clinical training would better prepare future doctors to practise safely and effectively. A practical approach may be providing protected time for medical students during their clinical placements to engage with HIS in a well-integrated manner, allowing them to develop their skills before graduation. Such exposure could help students develop transferable health competencies within a real-world clinical environment, although further work is required to determine the most effective learning outcomes, educational methods, and approaches to competency assessment.
